# The Effect of Self-Care Education on the Awareness, Attitude, and Adherence to Self-Care Behaviors in Hospitalized Patients Due to Heart Failure with and without Depression

**DOI:** 10.1371/journal.pone.0130973

**Published:** 2015-06-19

**Authors:** Ali Navidian, Fariba Yaghoubinia, Alireza Ganjali, Sadegh Khoshsimaee

**Affiliations:** 1 Pregnancy Health Research Center, Zahedan University of Medical Sciences, Zahedan, Iran; 2 Faculty of Medicine, Zahedan University of Medical Sciences, Zahedan, Iran; 3 Faculty of Nursing and Midwifery, Zahedan University of Medical Sciences, Zahedan, Iran; University of Bologna, ITALY

## Abstract

**Background:**

Cardiovascular diseases are among somatic disorders and psychological factors affect their onset, exacerbation, and treatment. This study was conducted on the hospitalized patients who had heart failure with and without depression. The study criteria was to evaluate the effect of self-care education on awareness, attitude, and adherence to self-care behaviors on these patients.

**Materials and Methods:**

In this quasi-experimental study, seventy patients with heart failure that met the inclusion criteria were recruited through purposive sampling method. They were assigned in to two equal size groups regarding their depression status. First, the eligible patients were selected; then Beck Depression Inventory was done on the patients followed by examination by the clinical psychologist. Patients with average and higher scores were classified in the depressed group and others who got lower than average scores were classified as the non -depressed group. A questionnaire containing items related to awareness, attitude, and adherence to self-care behaviors was used to collect the data. First, self-care behavior was determined and then a four-sessions of educational intervention were held individually for both groups. The second round of questionnaires were completed at patients’ home twelve weeks after the discharge. The Collected data was analyzed using independent-samples and paired-sample t tests, Chi square, and statistical analysis of covariance (ANCOVA) tests through SPSS (version 21, SPSS Inc., Chicago, IL, USA).

**Results:**

After the educational sessions, the statistical analysis showed significant differences in the mean scores of awareness, attitude, and adherence to self-care behaviors between the two groups (P<0.0001).

**Conclusion:**

Self-care behavior education had lower effects on the depressed patients with heart failure. Therefore, before providing education for these patients, it is necessary to consider their psychological problems such as depression.

## Introduction

Heart Failure (HF) is the most common cause of death in many countries [1]including Iran[2]Cardiovascular diseases (CVD) are the main cause of deaths worldwide. In developed countries, mortality rate due to CVD is 50% [3] and medical and nursing cares, hospital, drugs, and facility’s costs and also disability resulting from CVD seems to be the significant factors in this regard [4]. Similar to the other countries, the rate of HF is increasing in Iran; CVD was the main cause of death in 46% of cases in recent years regard[5].

In spite of advancements in medical sciences, the rate of death from HF is still high [6].The prevalence and incidence of CVD have significantly increased in the past five years [7]. Meanwhile, with regard to the aging population and improvement in the rate of survival among patients with myocardial infarction, the number of patients with CVD is expected to increase[8]. According to the statistics data from Iranian Center for Disease Control (2009), the rate of HF was 3.3% in 18 provinces of Iran[9].

In recent years, researchers have focused on clinical strategies and healthcare services to treat diseases associated with multiple health problems[10]. Anxiety, depression, anger, and lack of social supports are among the main psychological problems that patients with HF are dealing with[11].

Anxiety and depression with a chronic disease such as HF cause disability, which in turn increases medical costs, patient’s need for medical services and rate of mortality; however it decreases their QOL[12–13].

Educational programs such as stress management trainings, exercises and physical activities, nutrition, and healthy lifestyle could control the diseases efficiently[14]Several studies have reported that between 25% and 35% of patients with chronic diseases lack interest in training programs. Socio-psychological factors such as dependence, depression, and anxiety are among the factors affecting participation of patients in training programs[15].

Pozuelo et al. (2009) stated that depression and HF have a sophisticated association, which can be considered as causal relationship. In other word, depression is a risk factor of heart diseases and is associated with complications and death[16]. A higher rate of mortality was reported among the patients with myocardial infarction; these patients received higher scores in Beck Depression Inventory (BDI) test during a five-year follow-up program[17]. Depression may also cause high rate of mortality and disability among patients with coronary artery bypass grafting[18], congestive HF[19]and heart transplant[20].

Depression is an inhibiting factor in treatment of CVD since it causes denial of disease, low motivation to follow treatment[21],prolonged diseases, interference with the treatment and care, delayed recovery, and frequent hospitalizations[22]. In addition, depressed patients with CVD have lower self-care ability in comparison with non-depressed ones. Depressed patients have negative feelings about their physical health status and QOL because they believe that depression and low capacity of physical activity are associated with worsening the symptoms of the CVD[23]which decreases their QOL[24,31–25].Therefore, depression reduces the commitment of patients to medication regimes[26]and it is not surprising if depressed patients with HF have no commitment to therapeutic recommendations such as diet modification and lifestyle changes such as exercise and physical activity, smoking cessation, and participation in rehabilitation and educational programs[27]. Depression also reduces the efficacy of self-care training programs[10].

Self-care is one of the important modalities to control heart diseases[28]. So in these patients, adherence to self-care behaviors is of great importance[29]. Improper self-care behaviors may results in poor health outcomes[30]and cause frequent hospitalizations[31]. Studies have indicated that at least 50% of patients with HF do not adhere to medical recommendations, which results in frequent hospitalizations[32]. In contrast, proper self-care behaviors are an important factor to promote positive health outcomes and prevent frequent hospitalizations[33]. Hence, in recent years, the treatment mostly focused on compliance, training, and self-care[34].Depression and lack of adherence to medical instructions in patients with CVD have negative synergistic effects and worsen patients’ situation[35].

Evidences have shown that the effect of psychological variables on the rate of mortality and disability of patients with CVD is at least equal to clinical and demographic variables. Although, few studies concerning the effect of psychological factors on the psychological complications of CVD have been conducted in Iran. Lack of complete understanding of the role of psychological factors in the progression of CVD is possibly one of the reasons for high rate of mortality and disability among these patients. Unfortunately, in Iran and especially in less developed regions, patients with HF have limited access to the comprehensive cardiac rehabilitation programs and most of the trainings are provided during hospitalization. These simple educational programs do not focus on the role of psychological factors such as depression. Therefore, the current study aimed to evaluate the effect of self-care trainings on depressed and non-depressed hospitalized patients with heart diseases. In addition, the current study aimed to evaluate the negative effects of depression on the level of learning self-care behaviors in patients with HF.

## Materials and Methods

This quasi-experimental study was conducted on patients with HF who were hospitalized in CCU and post-CCU wards of educational hospitals affiliated with Zahedan University of Medical Sciences in 2013. Based on similar studies, the sample size was estimated to be 70 subjects, with 95% confidence interval and the power of 0.9. The participants were split randomly into two equal groups, During April to October 2013, purposeful method was used for hospitalized patients’ continuous recruitment. The most important inclusion criteria were as follow: a definitive diagnosis of HF by cardiologist, ejection fraction ≤ 40%, residing in Zahedan city, Iran, accessibility for further follow-up, being at least 20 years old, ability to write and read, have full consciousness and orientation. On the other hand, patients with difficulty in hearing and speaking, having less than one week to discharge program, physical disability, dialysis, and previous participation in rehabilitation programs were excluded.

### Instruments

Self-Care Behaviors Questionnaire: A primary questionnaire was designed by searching related references, textbooks and results of the previously completed studies. As a trial, limited number of hospitalized patients with HF were asked to complete the questionnaire to determine the clarity of the questionnaire items. During follow-up interview, patient’s comments were collected reflecting the ambiguities in the trial version of the questionnaire. Based on the received comments and recommendations, trial questionnaire was modified. Ten CCU nurses, health educators, and two cardiologists reviewed the questionnaire’s contents for structural and content validity after addressing all the comments, questionnaire’s content validity rating (CVR) and content validity index (CVI) were calculated and were determined to be at 0.71 and 0.81, respectively. Internal consistency method was used to determine the reliability of the questionnaire. For determining the stability of the questionnaire, test-retest method was applied. At first, a primary test was performed on 20 hospitalized patients with HF in the CCU and PCCU wards who were not enrolled in this research. In second, the questionnaire was completed after ten days again. At third stage, two questionnaires were compared using correlation tests with Cronbach's alpha. At fourth stage, the items with correlation coefficient of lower than 0.7 were omitted. The questionnaire verified ultimately. The mean Cronbach's alpha scores were calculated and in awareness, attitude, and adherence to self-care behaviors were 0.81, 0.87, and 0.81. Also the correlation coefficient were 0.77, 0.86 and 0.87, respectively.

The final questionnaire contained four parts. Part A which contained nine items about demographic data of the patients. Part B consisted of 13 items about the level of awareness regarding self-care behaviors. True answers had three scores, “I don’t know” had two scores, and false answers had one score (total score range, 13–39). If the subject responded “I don’t know”, it meant she/he was ready to be trained, but if the subject responded “false”, It meant that the subject’s wrong behavior had to be eliminated before training the right behavior. Part C had 12 five-choice items about attitude toward self-care behaviors. The options were: “totally agree”, “I agree”, “have no idea”, “I disagree”, and “totally disagree”, which were scored from one to five (total score range, 12–60). Part D of the questionnaire was derived from European Heart Failure Self-Care Behavior scale (EHFSCB). It consisted of 12 five-choices: “always”, “often”, “sometimes”, “rarely”, and “never”). They scored from one to five (total score range, 12–60) and, related data about adherence to self-care behaviors.

Beck Depression Inventory: This questionnaire is widely used to evaluate the level of depression among both psychiatric and non-psychiatric patients such as patients with HF(16). BDI is a self-report 21-item questionnaire. Each item is scored from zero to three. Internal consistency coefficient varies from 73 to 92 with a mean score of 86. Concurrent validity with clinical rating for psychiatric patients indicated moderate to high correlation coefficient (55% to 96%). Different conducted studies have approved validity and reliability of BDI. Ghasemzadeh et al. (2005) also calculated the content validity and reliability of BDI 0.87 and 0.74 in Iran. Score zero through nine indicate non depressed and normal mood, ten through 16 indicate mild, 17 through 20 moderate, 21 through 30 severe, and more than 30 indicate very severe depression[36].

At First, patients who met inclusion criteria, i.e., those who were hospitalized in CCU and post-CCU wards, were selected from April to October 2013. The selected patients were examined by a clinical psychologist immediately after admission in the ward and level of depression was recorded through BDI and diagnostic interview based on Mental Status Examination (MSE). The subjects were assigned to either depressed patients or non-depressed patients groups. This procedure was continued to saturate the sample size. Patients were homogenized in both groups in terms of sex, age, education, and marital status. Then the self-care behavior questionnaire was distributed to the subjects for completion by the subjects themselves.

In Second, the educational intervention was provided for both groups in four sessions (each session lasted for one hour) through individually face-to-face training program using an educational booklet and CD during the last days of hospitalization. This intervention have been developed, used and tested in Iran by Seraji et al. 2013(Table 1). Twelve weeks after discharge, the self-care behaviors questionnaire was filled out again by the patients of both groups at their homes. In case of inaccessibility or patient’s death, another patient was substituted.

**Table 1:** Structure and Concept of Educational Sessions for the Patients with Heart Failure.

Sessions	Educational Concept
First	Definitions of heart failure, diseases symptoms, and causes
Second	The importance of self-care, diet, weight control, resting and activity, and measurement of urine volume
Third	Monitoring worsening symptoms and necessary actions, vaccination, restricted alcohol consumption and smoking cessation, and importance of drug consumption
Fourth	Short movie viewing, question and answer regarding the raised issues, and conclusion

### Main Hypothesis

The main hypothesis in this study is “whether the impact of education on self-care behaviors of depressed and non-depressed patients is the same or different?”

### Data Analysis

The data were entered into a database using Statistical Package for the Social Sciences version 21.0 (SPSS Inc, Chicago, IL,USA). All of the comparisons were two-tailed and P-value of less than 0.05 were considered as significant in this study. Descriptive statistic such as percentages, mean, standard deviations were used to describe the sample on the various variables. To compare some variables between the control and intervention groups, independent t test (for quantitative variables) and Chi-Square test (for qualitative variables) was used. Analysis of covariance test was used to test the effect of self-care education in two groups. The effects of some confounding variables such as age, gender, marital status, level of education and job were covariate.

### Ethical Considerations

The current study was approved by the Committee of Ethics of Islamic Azad University of Zahedan, Iran, in 2013. Ethical considerations of the study contained applied methods and tools, the aim of study, duration of patients’ intervention, obtaining verbally informed consent, confidentiality of information, and withdrawal from the study at will. The participants in this research provided oral informed consent and a check mark in questionnaire captured their agreement for participation. The study continued on oral consent basis as signature could have compromised the anonymity of the subjects therefore jeopardize the participation Some subjects who could have been belonging to a group of asylum seekers, may feel threatened by such requests to identify themselves on paper (Barbour 2008). The Committee of Ethics at Islamic Azad University of Zahedan approved and confirmed recruitment of this type of consent.

## Results

The participants’ age ranged from 38 to 67 years with the mean age of 57.37± 6.18 and 57.08± 5.89 years in the depressed and non-depressed groups, respectively. Women comprised more than 60% of the participants in each group. Most of the participants had low level of education in both groups. More than 90% of subjects were married in the both group. Since most of the subjects were women, the most frequent occupation was housekeeping. No significant statistical difference was observed between groups with regard to demographic characteristics Table 2.

**Table 2:** Demographic Characteristics of Depressed and Non-depressed Patients.

variable	Depressed	Non Depressed	Test results
	N (%)	N (%)	
**Sex**			
Women	22 (62.8)	21 (60)	P = 0.06
Men	13 (37.2)	14 (40)	
Total	35 (100)	35 (100)	
**Education**			
Primary	25 (71.4)	23 (65.7)	P = 0.3
Lower than diploma	6 (17.1)	8 (22.8)	
Higher than diploma	4 (11.5)	4 (11.5)	
Total	35 (100)	35 (100)	
**Marital**			
Single	2 (5.7)	3(8.5)	P = 0.8
Married	33 (94.3)	32 (91.5)	
Total	35 (100)	35 (100)	
**Occupation**			
Housekeeper	14(40)	13 (37.1)	P = 0.8
Employee	9 (25.7)	7 (20)	
Others	12 (34.3)	15 (42.9)	
Total	35 (100)	35 (100)	
**Main Complaint**			
Shortness of breath	23(65.7)	26 (74.2)	P = 0.5
Limbs swelling	8 (22.8)	7 (20)	
Cough and tiredness	4 (11.5)	2 (5.8)	
**Age**	Mean±SD	Mean±SD	P = 0.7
	57.37 ± 6.18	57.08 ± 6.89	

The most common complaint was the shortness of breath in both groups. Hypertension, diabetes, heart attack, and pulmonary diseases with the prevalence of respectively 74%, 52%, 48.6%, and 34% were the most common underlying diseases.

Independent-samples t test showed that there was not a significant difference in the mean score of awareness, attitude, and self-care behaviors between groups before the intervention (P<0.05). However, the mean score of awareness of non-depressed HF patients 21.93 ± 2.34 was significantly more than that of depressed HF patients 12.10 ± 3.70 (P< 0.0001) after intervention Table 3. The mean score of attitude of the non-depressed HF patients 49± 2.42 was significantly more than that of depressed patients 31 ± 2.76(P< 0.0001). Also the mean score of self-care behaviors in depressed HF patients 27 ± 4.54 was significantly lower than that of non-depressed HF patients 46 ± 3.81(P< 0.0001). In general after the intervention, the difference in the mean score of aforementioned variables was statistically significant between groups, and non-depressed patients with HF had significantly higher scores than depressed ones (P = 0.0001).

**Table 3:** Awareness, Attitude, and Adherence to Self-Care Behavior Scores in Depressed and non depressed Patients Before and After the Intervention.

Variable	Before	After	Paired t test
	Mean±SD	Mean±SD	
**Awareness**			
Depressed	8.07 ± 3.4	12.10 ± 3.7	0.01
Non depressed	8.23± 3.49	21.93 ± 2.3	0.0001
Independent t test	0.7	0.0001	
**Attitude**			
Depressed	29 ± 1.68	31 ± 2.76	0.1
Non depressed	31 ± 2.21	49 ± 2.42	0.0001
Independent t test	0.06	0.0001	
**Adherence**			
Depressed	24 ± 3.29	27 ± 4.54	0.3
Non depressed	26 ± 2.76	46 ± 3.81	0.0001
Independent test	0.5	0.0001	

In the depressed HF patients group the score of awareness, attitude, and performance of self-care behaviors before intervention was 8.07 ± 3.45, 29 ± 1.68 and 24.00 ± 3.29, respectively and after intervention was 12.10 ± 3.70, 31.00 ± 2.76 and 27.00 ± 4.54, respectively. The results of the paired-sample t test showed an increase in the mean score of awareness after intervention in the depressed group (P = 0.01) while insignificant changes was observed in the other two variables, namely, attitude and adherence to self-care behaviors, after the intervention.

However, in non-depressed HF patients group the mean score of awareness, attitude, and performance of self-care behavior before intervention was 8.23 ± 3.49, 31.00 ± 2.21 and 26.00 ± 2.76 respectively and after intervention increased to 21.93 ± 2.34, 49.00 ± 2.42 and 46.00 ± 3.81, respectively. In non-depressed HF patients group, the mean scores of all three mentioned variables significantly increased after intervention (P<0.0001) Table 3.

Results of ANCOVA, with elimination of pretest and also the effect of some demographic characteristics such as age, sex, marital status, education, and occupation, showed that the effect of self-care behaviors education program on increasing the mean score of awareness, attitude, and adherence to self-care behaviors was higher in the non-depressed patients than in the depressed ones Tables 4–6.

**Table 4:** The Mean Scores of Pretest and Posttest of Awareness about Self-Care Behaviors in Study Groups, Based on ANCOVA Results.

Variable	Sum of squares	Degree of freedom	Mean square	F	Level of significance	Level of impact	Test Power
Pretest	49.61	1	49.61	5.081	0.02	5.08	0.60
Age	1.21	1	1.21	0.125	0.72	0.12	0.06
Sex	0.48	1	0.48	0.050	0.82	0.05	0.05
Marital	96.28	1	96.28	9.856	0.01	9.85	0.87
Education	26.28	1	26.28	26.282	0.10	26.28	0.36
Occupation	0.07	1	0.07	148.332	0.006	148.33	0.05
Group	1436.30	1	1436.30	147.368	0.0001	147.36	1.00
Error	632.35	63	9.76				
Total	275893	70					

**Table 5:** The Mean Scores of Pretest and Posttest of Attitude toward Self-Care Behaviors in Study Groups, Based on ANCOVA Results.

Variable	Sum of squares	Degree of freedom	Mean square	F	Level of significance	Level of impact	Test Power
Pretest	2.27	1	2.27	0.40	0.52	0.40	0.90
Age	0.16	1	0.16	0.03	0.86	0.03	0.05
Sex	1.73	1	1.73	0.31	0.57	0.31	0.08
Marital	2.57	1	2.57	0.45	0.50	0.45	0.10
Education	1.73	1	1.73	0.30	0.58	0.30	0.08
Occupation	12.25	1	12.25	2.18	0.14	2.18	0.30
Group	3716.60	1	3716.60	663.21	0.0001	663.21	1.00
Error	347.44	63	5.604				
Total	127693.00	70					

**Table 6:** The Mean Scores of Pretest and Posttest of Adherence to Self-Care Behaviors in Study Groups, Based on ANCOVA Results.

Variable	Sum of squares	Degree of freedom	Mean square	F	Level of significance	Level of impact	Test Power
Pretest	12.05	1	12.05	0.94	0.33	0.94	0.15
Age	4.51	1	4.51	0.35	0.55	0.35	0.09
Sex	41.27	1	41.27	3.22	0.07	3.22	0.42
Marital	1.78	1	1.78	0.14	0.71	0.14	0.06
Education	13.54	1	13.54	1.05	0.30	1.05	0.17
Occupation	31.03	1	31.03	2.42	0.12	2.42	0.33
Group	4447.19	1	4447.19	347.08	0.0001	347.08	1.00
Error	794.42	63	12.81				
Total	155988.00	70					

## Discussion

Different factors such as factors related to patients, healthcare providers, and nature of the therapeutic regimen have led in poor adherence to treatment in the patients with HF[37].In the current study, It was investigated only the role of depression as one of the psychological factors.

Results of the current study showed that the effect of training was not similar in depressed and non-depressed patients with HF. In addition, the mean scores of self-care behaviors such as awareness, attitude, and adherence to self-care behavior were significantly higher in non-depressed than in depressed patients after intervention. The results of the current study was consistent with those of other studies conducted in Iran by Shojaeifard[38],Seraji[39]and MangolianShahrebabaki[40] and international studies conducted in other countries such as studies by Jaarsma[41],Scotto[42],Wang[43]and Krumholz[44]. All performed studies showed that educational or supportive interventions might improve self-care behaviors, promote QOL, and improve mental status of patients with HF regardless of the subject depression status. Vanderwal et al. (2006) demonstrated that adherence of patients with HF to some aspects of self-care behaviors is poor. He believed that increasing awareness, changing attitude and beliefs, and adhering to self-care behaviors through education and consultation is necessary, especially in depressed patients[37].

After intervention, the mean score of awareness increased in depressed patients with HF. However, there was no significant change in the mean scores of attitude and adherence to self-care behaviors. These results indicated that increasing awareness alone would not result in commitment to therapeutic regimen [37]. According to Health Belief Model (HBM), attitude and beliefs are the determinants of healthy behavior. The important construct of this model is perceived benefits and barriers of the treatment regimens. Belief in the effectiveness of the designed strategies for reducing the threat and disease is the component of perceived benefits. Also, the perceived barriers including the expectation of negative consequences arising from the use of hygienic practices. Due to disappointment, irrational beliefs, negativity, bipolar thinking pattern, catastrophic thinking, and cognitively impaired vision, depression prevents significant changes in the attitude, awareness, and self-care behaviors of patients with HF.

The result of the current study showed that education had no effect on the promotion of self-care behaviors in non-depressed group in comparison with depressed one. This result was compatible with those of other similar studies [39]. Results of Nakao et al. reported that patients who had received lower scores in The Symptom Checklist-90 (SCL-90), i e, had lower psychological symptoms, were more likely to complete the therapeutic program in comparison with the group who had more psychiatric symptoms[14]. Moreover, another study showed that the risk of low adherence to drug consumption in depressed and anxious patients with HF was 4.4 times as high as non-depressed ones[45].Caseyet al. showed that depression might result in failure to complete the rehabilitation program in patients with HF. Logistic regression of this study indicated that patients with HF who received scores>10 in BDI usually fail to complete 12-week rehabilitation program 2.2 times as more as patients who received scores of less than ten[46].Study of Coyle also indicated that due to symptoms of restlessness and low energy, depression after heart attack significantly affected adherence to self-care behaviors after discharge[47].Depression symptoms may cause poor and insufficient adherence to the consumption of aspirin in acute coronary syndrome[48]. Low adherence to smoking cessation, drug consumption, regular exercise, and participation in therapeutic sessions.

Depression not only affects patients with HF but also may affect commitment to the therapeutic programs in other patients with chronic diseases. In a meta-analysis study by Grenard et al. on depression and adherence to drug consumption in chronic diseases,31 studies including18245 participants in the United States were evaluated. Results of the study showed that the level of commitment to drug consumption in depressed patients was 1.76 times lower than the non-depressed patients[49].

Psychological symptoms inhibit self-care behaviors in patients. They often cannot properly learn how to change their lifestyle and usually do not follow recommended medical prescriptions, activities, and nutritional diet[50].

According to Diagnostic Statistical Manual of Mental Disorders (DSM IV-TR), depressed patients with HF experience a set of emotional (eg, sorrow and grief), physical (eg, tiredness and powerlessness), and cognitive (eg, difficulty concentrating) symptoms [51]. Disorder in learning self-care behaviors may result from depression complications such as difficulty in concentrating, low motivation, low ability in cooperation with others, sense of purposelessness, tiredness, physical pains, low energy, lack of effort to achieve the goals, inability to make decisions, anger, feelings of worthlessness and guilt, and lack of interest in work and activities[10,47,52,53]. A Study by DiMatteo, Lepper&Croghan showed that depression was an important factor in poor adherence to therapeutic regimes. They believed that mood disorders might affect motivation and ability of patients with HF to follow therapeutic and educational programs. Social isolation and reduced cognitive function in depression might negatively affect learning and commitment to programs[54].Ciechanowski reported that depressed patients with HF often know what they have to do to improve their situation, but depression prevents them and their emotional mind overcomes their rational mind[10]. Depression increases the possibility of improper habits adoption such as emotional eating, physical inactivity, and smoking. On the other hand, poor adherence to therapeutic programs in association with lack of self-care may disappoint patients’ expectations in having control over their diseases. Therefore, the result of the current study recommends providing better strategies to manage depression, which is the most important psychological problems, and to decrease its complications in patients with HF[50].

### Limitations

Lack of control group without any educational intervention, low sample size, history of hospitalization, impact of previous self-care trainings, and short-term education during hospitalization are among the limitations of the current research. It is recommended that the broader study concerning the cardiac rehabilitation program should be done for patients who are hospitalized for the first time. Then; it will help to overcome the limitations of current study.

## Conclusion

Considering the high prevalence of depression and its symptoms in patients with HF, results of the current study revealed that the effect of self-care training in the depressed patients with HF is lower than non-depressed ones. It means that depression and its relevant symptoms act as a psychological obstacle to learning self-care behaviors in patients with HF. Lack of commitment to the self-care behaviors leads to worsened mental and physical status, frequent hospitalizations, higher rates of complications, and increased rate of mortality among patients. Hence, it is necessary to screen patients with HF from the viewpoint of depression via questionnaires (American Heart Association Questionnaire, AHA) or diagnostic interviews; moreover, short-term as well as long-term treatment of symptoms of depression by a clinical psychologist should be considered. The most important point is that the designed self-care programs should be flexible enough for CCU nurses to meet psychological treatment requirements (e g, cognitive behavior therapy), services, and consultations (e g, motivational interviewing), or to be used as a pre-treatment to increase the efficacy of therapy.
